# Dual fluoroscopic imaging and CT-based finite element modelling to estimate forces and stresses of grafts in anatomical single-bundle ACL reconstruction with different femoral tunnels

**DOI:** 10.1007/s11548-021-02307-2

**Published:** 2021-01-20

**Authors:** Yang Xiao, Ming Ling, Zhenming Liang, Jian Ding, Shi Zhan, Hai Hu, Bin Chen

**Affiliations:** 1 Division of Orthopaedics and Traumatology, Department of Orthopaedics, Nanfang Hospital, Southern Medical University, No. 1838 North Guangzhou Avenue, Guangzhou, 510515 Guangdong China; 2 Department of Orthopedics, Academy of Orthopedics Guangdong Province, Guangzhou, China; 3 Department of Orthopaedics, Fudan University Affiliated Huadong Hospital, Shanghai, China; 4 Department of Orthopedic Surgery and Orthopedic Biomechanical Laboratory, Shanghai Jiao Tong University Affiliated Sixth People’s Hospital, No. 600 Yishan Road, Shanghai, 200233 China

**Keywords:** Finite element analysis, Fluoroscopy, Anterior cruciate ligament, Femoral tunnel, Force, Stress, In vivo

## Abstract

**Purpose:**

Little is known about the in vivo forces and stresses on grafts used in anterior cruciate ligament (ACL) reconstruction. The aims of this study were to evaluate and compare the forces and stresses on grafts used in anatomical single-bundle ACL reconstruction at different locations of the femoral footprint (anterior vs middle vs posterior; high vs middle vs low) during a lunge motion.

**Methods:**

Establish subject-specific finite element models with different graft’s tunnel loci to represent the primary ACL reconstructions. A displacement controlled finite element method was used to simulate lunge motions (full extension to ~ 100° of flexion) with six-degree-of-freedom knee kinematics data obtained from the validated dual fluoroscopic imaging techniques. The reaction force of the femur and maximal principal stresses of the grafts were subsequently calculated during knee flexion.

**Results:**

Increased and decreased graft forces were observed when the grafts were located higher and lower on the femoral footprint, respectively; anterior and posterior graft placement did not significantly affect the graft force. Lower and posterior graft placement resulted in less stress on the graft at higher degrees of flexion; there were no significant differences in stress when the grafts were placed from 0° to 30° of flexion on the femoral footprint.

**Conclusion:**

The proposed method is able to simulate knee joint motion based on in vivo kinematics. The results demonstrate that posterior to the centre of the femoral footprint is the strategic location for graft placement, and this placement results in anatomical graft behaviour with a low stress state.

## Introduction

Anterior cruciate ligament (ACL) reconstruction is a technical procedure. Femoral tunnel positioning is one of the most critical steps to achieve successful ACL reconstruction and is still being discussed by researchers [1]. Because new anatomical findings of the ACL femoral footprint have emerged [2, 3], studies on anatomical ACL reconstruction have been conducted recently, and they have shown that anatomical graft placement is important to restore normal ACL function [4].

The graft force needs to be considered to restore normal ACL function. Measuring the forces on native ACLs continues to be one of the greatest challenges in assessing the biomechanics of the knee joint. To overcome this challenge, investigators have proposed a technique involving the isolation of the tibial attachment of the cruciate ligament by creating a bone plug and attaching a load sensor to measure the native ACL force in cadaveric knees [5, 6]. This approach is limited to ex vivo applications. Because forces and stresses are directly related to strains, investigators have measured ligament displacements and calculated strain patterns to gain an understanding of the in vivo biomechanics of ACL grafts recently [7]. The combined magnetic resonance (MR) and dual fluoroscopic imaging analysis is useful for in vivo applications. But strains were predictions from the measurements of length change and no actual ACL reconstructions were performed in their works. Others also characterize knee joint kinematic function in three-dimensional (3D) based on MR images under weight-bearing conditions or measure length of ligament using MR images combined with motion capture system, but no further biomechanical analysis was performed [8, 9]. Finite element analysis is a useful tool for clinicians to investigate biomechanics of the knee joint for it can simulate virtual surgical operations. However, the use of graft forces and stresses measurements to evaluate the physiologic weight-bearing state of the knee after ACL reconstruction has not been clearly reported in the literature.

Therefore, this study used displacement controlled finite element analysis combined with dual fluoroscopic imaging techniques to evaluate the effects of placing the graft at different locations on the femoral footprint after anatomical single-bundle ACL reconstruction via lunge simulations. The results from this study may help surgeons to gain a better understanding of the influence of grafts placed at different femoral tunnel locations in terms of the forces and stresses and contribute to the current literature by providing biomechanical information on ACL reconstruction.

## Materials and methods

### Subject recruitment

**Fig. 1:**
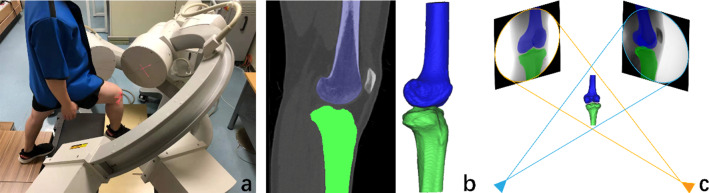
**a** Dual fluoroscopy was used to record the motion of the subject's right knee while he performed a lunge motion. **b** CT imaging was used to create 3D models of both the femur and tibia.** c** The 3D models were synchronized with the dual fluoroscopic images to reproduce the motion of the subject's knee during the movements

This study was approved by our institutional review board, and informed consent was obtained by the subject. One healthy volunteer (male; 26 years; body height 175 cm; body weight 65 kg) was recruited for this study. The subject had a right dominant limb and no history of knee injuries, surgeries or systemic diseases. Physical examinations (Lachman test and pivot shift test) were performed to rule out knee pathologies. The motion of the right knee was analysed (Fig. 1a).

### Imaging

Computed tomography (CT) and dual fluoroscopic imaging techniques have been described in detail previously [10, 11]. Similar methods to investigate in vivo knee kinematics have been used in our previous work [12, 13]. CT scans (SOMATOM Definition AS + ; Siemens) of the right knee joint ranging from approximately 15 cm proximal and 15 cm distal to the joint line (thickness, 0.6 mm; resolution, 512 × 512 pixels) were obtained. The images were then imported into Mimics (v19.0, Materialise NV, Leuven, BE). CT Bone Segmentation (threshold:1250–3680; fill holes) and Smoothing (iterations:7; smooth factor:0.7) were used to calculate 3D models of the femur and tibia. The CT-based models were exported in STL format (Fig. 1b). The knee of the subject was simultaneously imaged using two fluoroscopes (BV Pulsera; Philips) as the subject performed a lunge motion (full extension to ~ 100° of flexion). The cumulative radiation dose was 4.95 mGy during the whole test, which was less than that of a conventional abdominal CT scan. Considering that the target motion took only 5 s in the overall 32 s, it was possible that the radiation dose would reduce markedly when the procedure was maturely used. Next, the fluoroscopic images were imported into MATLAB (R2013a; MathWorks) and positioned in the imaging planes based on the projection geometry of the fluoroscopes when the subject was scanned. Finally, the STL models were imported into the software, viewed from the directions corresponding to the fluoroscopic X-ray source used to acquire the images, and manually manipulated in six degrees of freedom (6DOF) with the software until the projections of the model matched the outlines of the in vivo fluoroscopic images taken when the subject performed the knee motion (Fig. 1c).

### 6DOF calculation

**Fig. 2:**
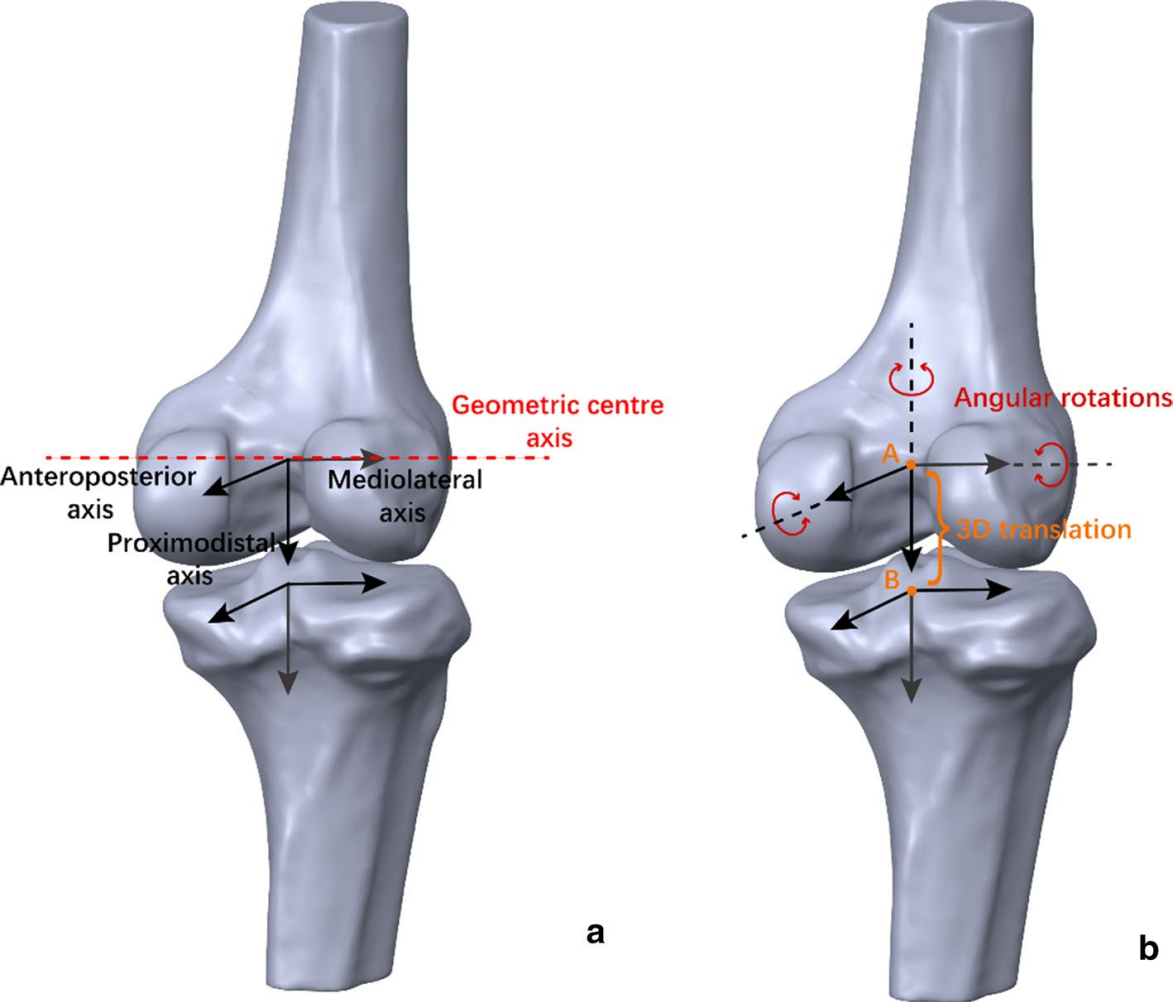
**a** Definitions of the local femoral and tibial coordinate systems. **b** Definitions of the Euler angles and 3D translation. *A* reference point of the femur; *B* reference point of the tibia. Three angular rotations and a 3D translation steps were performed with finite element analysis

**Fig. 3:**
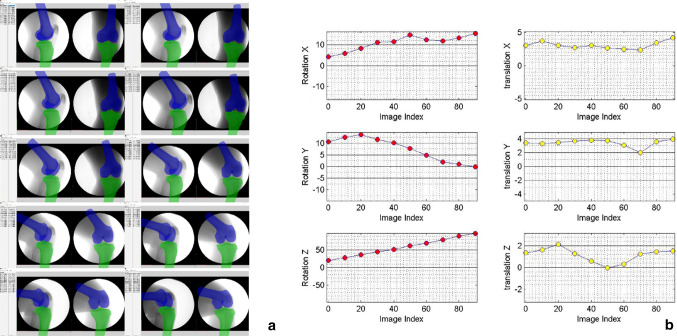
Screenshots of the alignment and the output of the results. **a** Projections of the models of each frame were matched. **b** 6DOF changes in the process of knee motion (ten points correspond to ten flexion angles from 10° to 100°)

Local femoral and tibial coordinate systems were established before the imaging procedure. The femoral origin was located at the midpoint of the geometric centre axis, a line connecting the centres of the spheres fit to the lateral and medial posterior femoral condyles, representing the mediolateral axis. The anteroposterior axis was perpendicular to the plane defined by the geometric centre axis and the femoral shaft. The proximodistal axis was set to be perpendicular to the two other axes. The tibial origin was the projection point of the femoral origin on the tibial plateau. In order to make the calculated kinematic data easier to be applied in the finite element analysis subsequently, the three axes of the tibial coordinate system were parallel to the femoral coordinate system (Fig. 2a). The 3D translation was quantified as the relative displacement between the origins of the femur in the tibial coordinate system. The angular rotations were determined by the femoral coordinate system with respect to the tibial coordinate system using Euler angles in the following sequence: mediolateral axis (Z axis), anteroposterior axis (X axis) and proximodistal axis (Y axis) [14]. The tibial coordinate system was defined as fixed coordinate system and the femoral coordinate system as floating coordinate system. After matching the projections of the models to their corresponding outlines, the rotation matrix of the floating coordinate system relative to the fixed coordinate system was calculated. Through the rotation matrix, the relative translation and rotation of the two coordinate systems can be calculated according to the sequence described above. The 6DOF in each frame were connected in series to form the 6DOF changes in knee flexion (Fig. 3).

### Surgical modelling

**Fig. 4:**
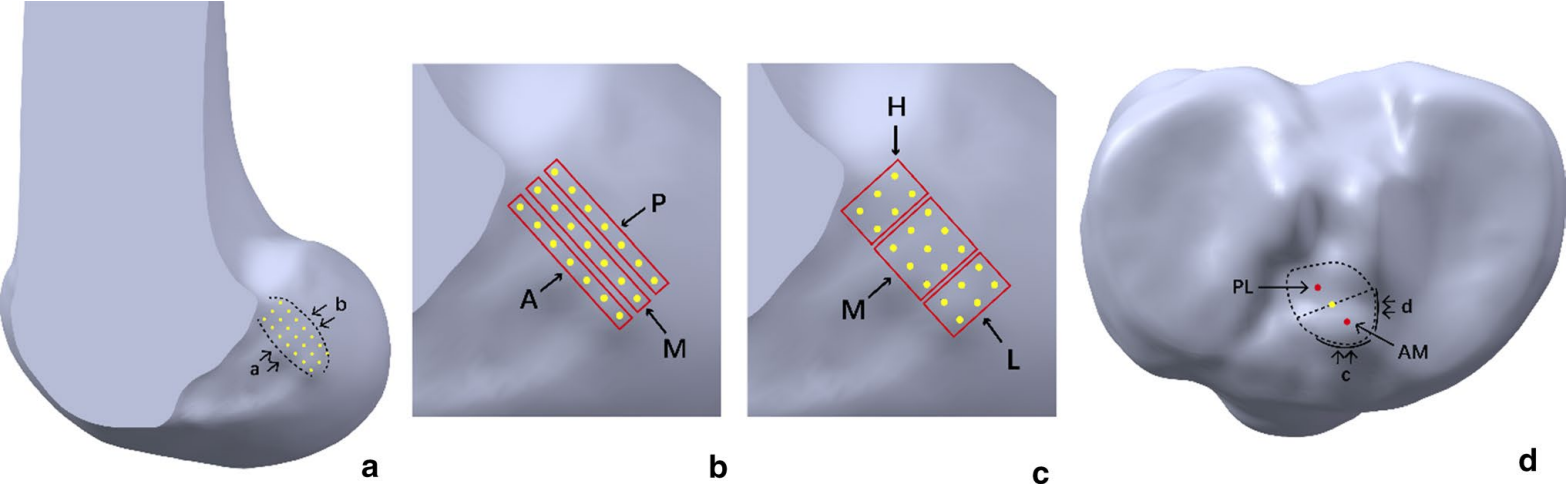
**a** The twenty-one femoral attachment points of the ACL on the femoral footprint. *a* Lateral intercondylar ridge; *b* the edge of femoral articular cartilage. **b, c** The different locations. *A* anterior location; *M* middle location; *P* posterior location. *H* high location; *M* middle location; *L* low location. **d** The tibial attachment point of the ACL that was identified based on the bony landmarks on the 3D CT models. *PL* posterolateral; *AM* anteromedial; *c* anterior ridge; *d* medial intercondylar ridge

**Fig. 5:**
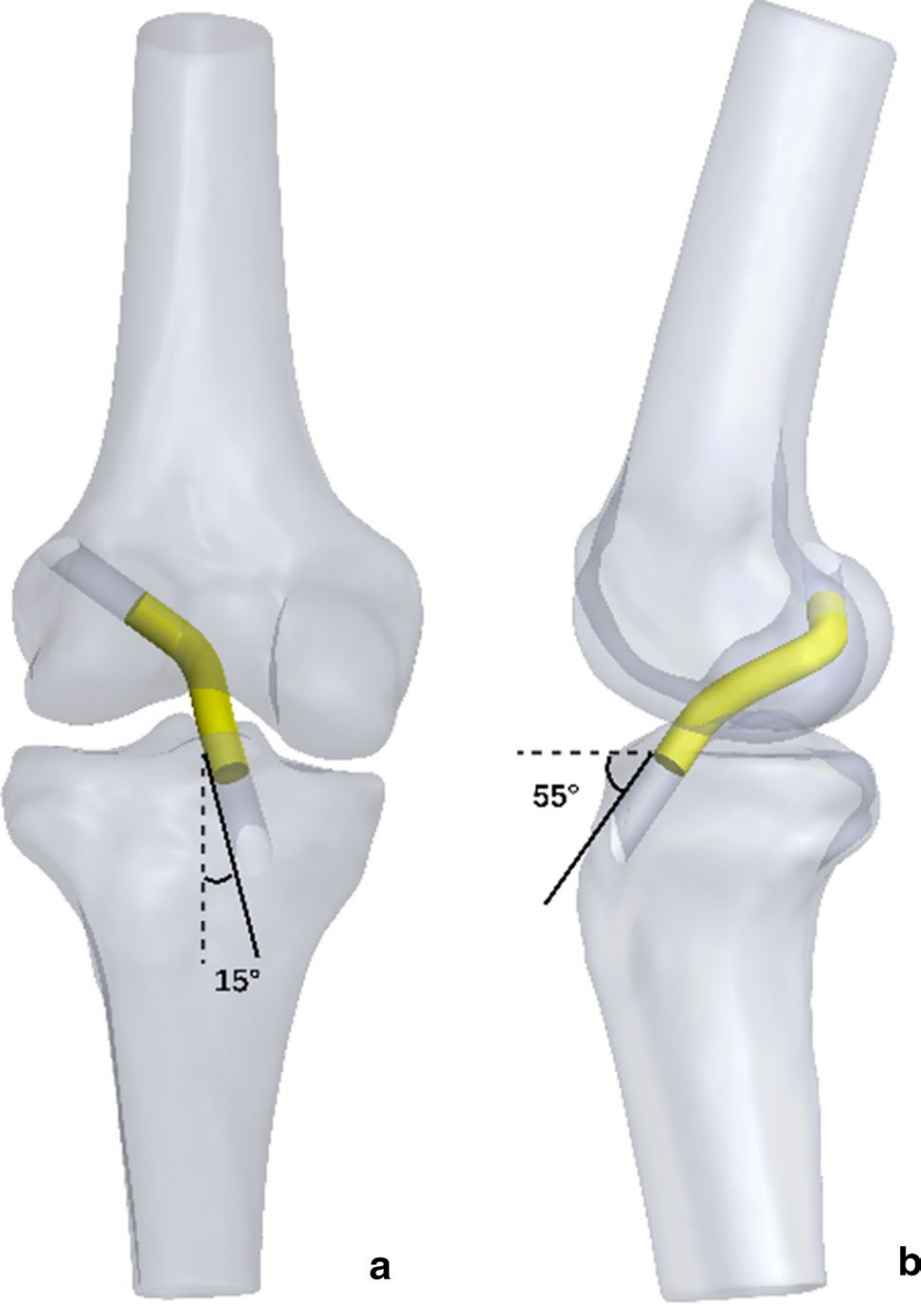
**a** Anterior view of the model at 20° of flexion. **b** Lateral view of the model at 20° of flexion

The femoral bony landmarks (lateral intercondylar ridge and the edge of femoral articular cartilage) were identified and used to determine the femoral footprint of the ACL [2, 3] (Fig. 4a). Twenty-one femoral attachment points were mapped to cover the footprint completely. Each point was 2 mm apart from the others. In addition, we categorized these points based on their location (anterior, middle and posterior; high, middle and low) (Fig. 4b, c). The tibial attachment point was identified as the midpoint of the line that connected the centres of the double bundle of the ACL on the tibial footprint [15] (Fig. 4d). Femoral tunnels were simulated using the anteromedial portal for each attachment point. The tibial tunnel was simulated at 15° in the anterior view and 55° in the lateral view (Fig. 5). The grafts measured 8 mm in diameter and 10 cm in depth into the femoral and tibial tunnels and were constructed as single, soft cylindrical solids. All the modelling procedures were performed using SolidWorks (v2018, Dassault Systemes, Massachusetts, USA).

### Finite element analysis

The bones were considered shells and defined as rigid bodies. The grafts were considered solids and were defined as nearly incompressible, transversely isotropic hyperelastic neo-Hookean materials with the following strain-energy function: $\Psi  = \frac{1}{{2D}}In(J^{2} ) + C_{1} (\overline{{I_{1} }} ^{2}  - 3) + F_{2} (\lambda )$, where *C*_1_ is the neo-Hookean constant and *D* is the inverse of the bulk modulus* k* = 1/*D* (C1 = 1.95, *D* = 0.00687) [16]. A free meshing technique was used for the grafts using eight-node linear hexahedral elements with hourglass control (e.g. element type C3D8RH). In order to achieve balance between the accuracy of stress and the computational time, a mesh sensitivity analysis was performed by stepwise upsizing the mesh size, according to a previous study [17]. The convergence tolerance was set as a variation of stress within 5% from the previous model with higher mesh density. As a result, the optimum mesh size of grafts was between 0.9 and 1.1 mm. The models of graft included an average of 6000-plus nodes with 5000-plus elements. Bonded contact between the graft and internal surface of the femoral tunnel and frictionless contact between the graft and tibial tunnel were defined. The femoral and tibial coordinate systems were established as described above. The reference points of the femur and tibia were their origins in the coordinate systems.

**Fig. 6:**
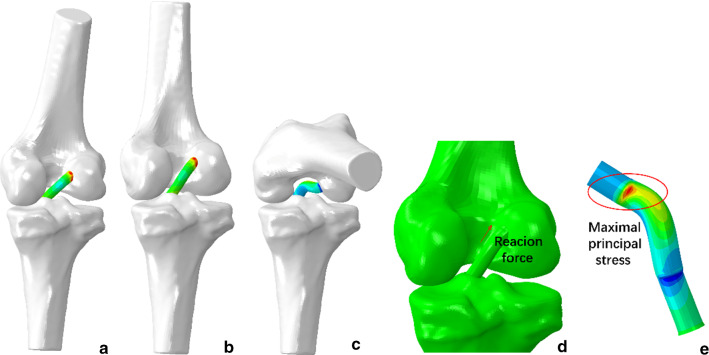
An example of the finite element analysis process with graft placement on the centre of the footprint is shown from **a – e. a** The model at 20° of knee flexion is shown after an initial graft tension of 80 N was applied. **b, c** The models at the initial and final positions (0° and 100°) are shown as examples.** d** The reaction force of the femur was calculated for each model during the entire range of motion.** e** The maximal principal stress on the graft was near the femoral tunnel

To replicate the ACL reconstruction completely, a model at 20° of knee flexion and an initial graft tension of 80 N were used [18, 19]. The boundary and loading conditions were implemented in two different steps to imitate the actual state of the knee after ACL reconstruction and during the knee lunge motion as the researchers did previously [15]. First, the femur and tibia were fixed, and an initial graft tension of 80 N was applied on the end surface of the graft along the tibial tunnel direction. Second, the tibia and the portion of the graft in the tibial tunnel were fixed, and the femur was translated three-dimensionally and rotated angularly according to the 6DOF calculation (Fig. 2b). Knee motion was simulated at each of the flexion angles and quasi-static simulation was performed. We simulated the motions by assuming a state. At a certain flexion angle, 6DOF was applied to the femoral model relative to the tibial model, causing the deformation of the graft model relative to the initial position. The femur position was simulated in 10° increments as outer loadings for the graft. The reaction force of the femur and maximal principal stress of the graft were subsequently calculated (Fig. 6). Finite element analysis was performed in Abaqus (v2018; Dassault Systèmes SE, Vélizy-Villacoublay, FR), executed on a 64-bit Windows operating system with Quad-core and eight-thread Inter Core platforms configured with 24 GB of RAM. Simulation required approximately 10 min at each knee flexion angle. Total required time for performing this analysis was about 35 h.

### Statistical analysis

Analysis of variance (ANOVA) with repeated measures and the least significant difference (LSD) post hoc tests were used to detect statistically significant differences in the data. The within-subject factor was defined as the flexion angle. The between-subjects factor was defined as the location. At each tested flexion angle, the data from different groups were skewed distributed. Thus, we used two nonparametric tests for the comparison, the Kruskal–Wallis and Mann–Whitney U nonparametric tests were used to assess the differences across the different locations and to compare between pairs of the three locations if significant effects were detected. All analyses were performed in SPSS (v19.0, IBM Statistics, New York, USA), and *P* < 0.05 was considered significant.

## Results

### Reaction force

**Fig. 7:**
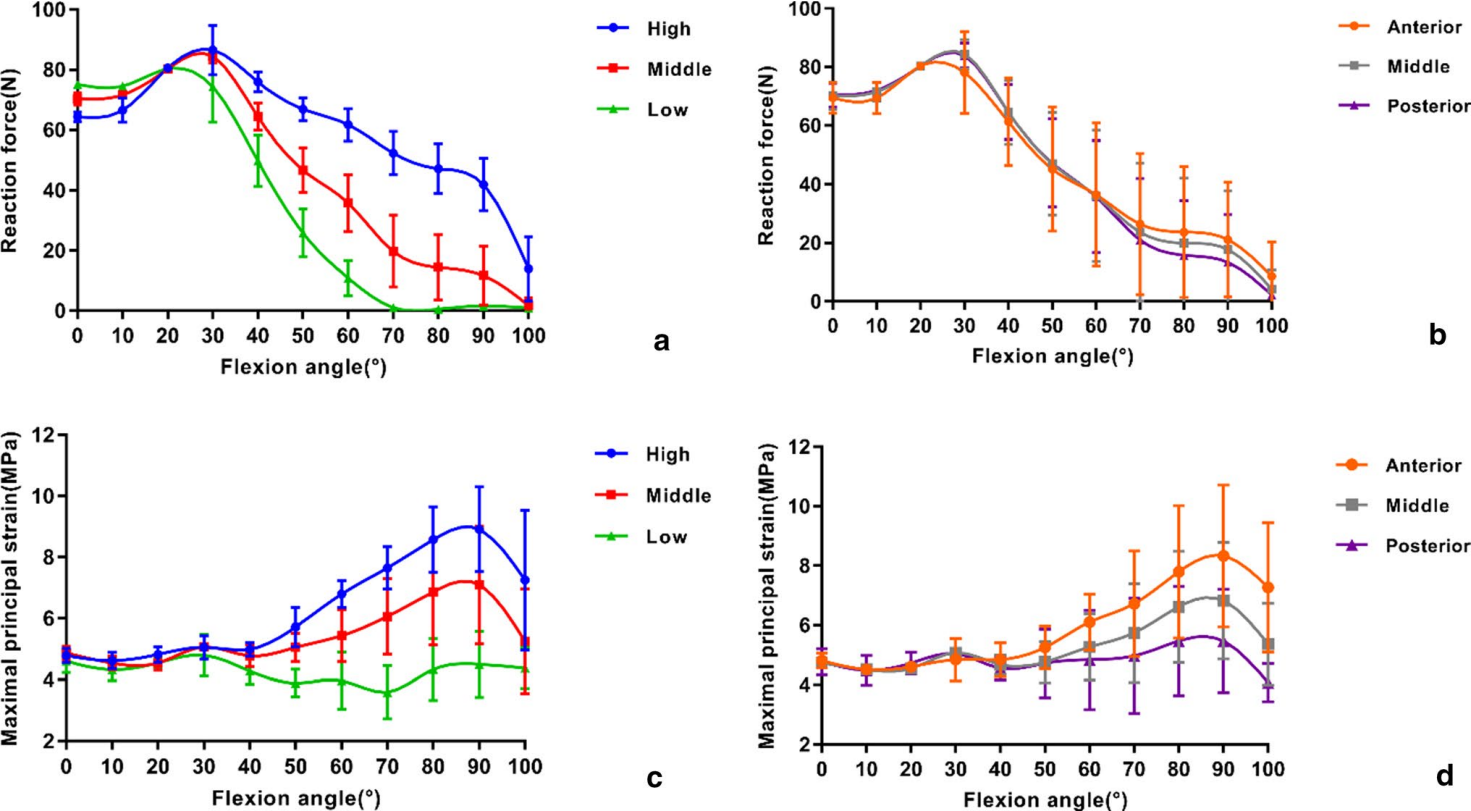
The data were compared in both the higher–lower and anterior–posterior directions. **a, b** Comparison of the reaction force at the different locations during the entire range of motion. **c, d** Comparison of the maximal principal stress at the different locations during the entire range of motion. All values are presented as the mean and standard deviation

The graft force first increased and then decreased after the maximum force was reached, which occurred at approximately 30° of flexion (Fig. 7a, b). The graft force was significantly affected by the graft placement locations in the higher–lower direction (*P* < 0.001). At high degrees of flexion, the high location led to a consistently larger force; the middle location led to anatomical graft behaviour similar to that of a normal knee, while the low location led the graft force to decrease early to nearly zero. No significant differences in the graft force were found when the graft was placed in the anterior–posterior direction (*P* = 0.971) (Table. 1A). At each tested flexion angle, significant differences were found among the high, middle and low locations (Table 2). The statistical results between pairs of the three locations are shown in Table 3a.

**Table 1:** Statistical analysis (ANOVA with repeated measures results) for the graft forces and stresses at the various locations (A) and between-group comparisons (LSD post hoc test results) (B)

(A)	Graft force		Graft stress	
	High vs middle vs low	Anterior vs middle vs posterior	High vs middle vs low	Anterior vs middle vs posterior
*P* value	< .001	.971	< .001	.104

(B)	High vs middle *P* value	High vs low *P* value	Middle vs low *P* value	Anterior vs middle *P* value	Anterior vs posterior *P* value	Middle vs posterior *P* value
Graft Force	< .001	< .001	< .001	.992	.831	.839
Graft Stress	.015	< .001	.003	.205	.037	.358

**Table 2:** Statistical analysis (Kruskal–Wallis nonparametric test results) for the graft forces and stresses at the various locations at each tested flexion angle

Flexion angle (°)	Graft force		Graft stress	
	High vs middle vs low *P* value	Anterior vs middle vs posterior *P* value	High vs middle vs low *P* value	Anterior vs middle vs posterior *P* value
0	< .001	.901	.257	.727
10	< .001	.610	.569	.977
20	.004	.955	.060	.529
30	.004	.834	.475	.532
40	< .001	.974	.035	.212
50	< .001	.996	.001	.219
60	< .001	.996	.001	.194
70	< .001	.884	.001	.224
80	< .001	.713	.002	.152
90	< .001	.732	.002	.074
100	.007	.599	.042	.003

**Table 3:** Between-group (the Mann–Whitney U nonparametric test results) comparison of the graft forces and stresses in the higher–lower direction (A) and graft stresses in the anterior–posterior direction (B) at different flexion angles

(A)	Graft force			Graft stress			
Flexion angle (°)	High vs middle *P* value	High vs low *P* value	Middle vs low *P* value	Flexion angle (°)	High vs middle P value	High vs low P value	Middle vs low P value
0	.001	.004	.001	40	.238	.016	.077
10	.002	.004	.002	50	.077	.004	.003
20	.001	.016	.858	60	.007	.004	.013
30	.045	.037	.001	70	.025	.004	.003
40	.001	.004	.001	80	.045	.004	.007
50	.001	.004	.001	90	.059	.004	.010
60	.001	.004	.001	100	.077	.016	.346
70	.001	.004	.001				
80	.002	.004	.002				
90	.002	.004	.002				
100	.007	.006	.812				

(B)			
Flexion angle (°)	Anterior vs middle *P* value	Anterior vs posterior *P* value	Middle vs posterior *P* value
100	.048	.003	.035

### Maximal principal stress

During the overall process of lunge simulation, the maximal principal stress of the graft appeared near the entrance to the femoral tunnel. The graft stress did not change significantly before 30° of flexion was reached. Afterwards, it first increased and then decreased after the maximum stress was reached, which occurred at 90° of flexion (Figs. 7c, d). Significant differences in the graft stress were found in the higher–lower direction (*P* < 0.001) but not in the anterior–posterior direction (*P* = 0.104) (Table 1A). However, significant differences in the graft stress were found between the anterior and posterior locations (*P* = 0.037) (Table 1B). The low and posterior locations showed lower levels of stress than the other locations. From 40° to 100° of flexion, significant differences were found among the high, middle and low locations. At 100° of flexion, significant differences were found among the anterior, middle and posterior locations (Table 2). The statistical results between the pairs of the three locations are shown in Table 3A, 3B.

## Discussion

Previously, researchers have used displacement controlled finite element analysis to study the biomechanics of ACL during knee flexion [15, 17, 20]. The applications of kinematics of the knee joint in these studies were not precise enough to reflect physiologic weight-bearing conditions. Using fluoroscopic imaging technique, the accurate in vivo kinematics of the bone structures can be obtained. The present study used CT and fluoroscopic imaging to derive subject-specific models and motions, and used finite element analysis to simulate ACL reconstructions. It showed a non-invasive way to assess the biomechanical status of ACL grafts in knee motion and hence has the potential to be used in patient-specific surgical planning and assessment.

In term of the model construction, most of the previous studies constructed the ACL graft as a single cylindrical solid [15, 21, 22]. In terms of material properties, there are roughly two types. One is fibre material, which takes into account the fibre direction and tensile stress–stretch relationship and is supposed to more accurately simulate the mechanical properties of the tendon; however, it needs laborious construction and complicated setting [23, 24]. For usage of an attentively designed fibre-dominated material, the help of skilled expert was required, and the research may take longer to complete than expected. The other is homogeneous material. Some researchers used homogeneous elastic materials to measure the reaction forces of the ligaments [25]. Considering the incompressibility of graft, a homogeneous hyperelastic material was used in this study. It simplified the model setting and mathematical processing and meanwhile guaranteed satisfactory results.

The most important finding of the present study is that the strategic femoral location for anatomical single-bundle ACL reconstruction is posterior to the centre of the footprint. These findings can be explained by the forces and stresses on the grafts. When restoring a normal ACL force–flexion curve, a lower level of stress on the graft is beneficial to prevent graft injury. Traditionally, the focus of ACL reconstruction has been on placing grafts in the most isometric manner, which can prevent the windshield wiper effect and is favourable for tendon-bone healing [26, 27]. A nonisometric graft can be expected to slacken during a large portion of the flexion cycle and to not prevent anterior translation [28]. However, the length of the native ACL was not isometric during knee flexion. A recent study highlighted the importance of restoring functional anatomy in ACL reconstruction to achieve normal knee function [4]. Isometric placement of the graft resulted in nonanatomical graft behaviour, which can overconstrain the knee at larger flexion angles [7]. Therefore, biomechanical considerations of grafts in ACL reconstruction are as important as isometric considerations are.

Multiple cadaveric studies have been conducted to investigate the graft force during passive flexion–extension. These studies used the force–flexion curve as an indicator to evaluate different ACL reconstruction techniques [29, 30]. It has been indicated that the placement of femoral grafts in different locations results in different graft forces [31]. In addition, studies demonstrated that passive flexion–extension motions do not load the ACL [5, 30], which is not completely consistent with the current results. In this study, the graft was loaded first and then was unloaded during the lunge motion, and the peak force occurred at 30° of flexion (Figs. 7a, b). Another experimental study simulated active extension of the lower limb against gravity by loading the quadriceps musculature [6]. The result showed an increased load on the ACL from 0° to 45° of flexion. These findings demonstrated the difference in graft behaviour between in vivo and in vitro conditions, which highlights the need for evaluating ACL reconstruction under physiologic conditions.

Large stresses on the graft and stresses close to the femoral or tibial tunnels were thought to be closely related to graft injuries and the widening of tunnels after surgery [15, 32]. Given the importance of avoiding high levels of stress, the results of this study seem to be significant. The posterior graft placement led to a lower level of stress during the lunge and was significantly different from the other locations at higher degrees of flexion, which was beneficial in reducing the graft stress and risk of injury. Regardless of the location at which the graft is placed on the femoral footprint, the maximal principal stress of the graft did not change obviously before 30° of flexion was reached. The peak stress was found at 90° of flexion (Figs. 7c, d). These findings may guide rehabilitation practice, during which a large range of knee flexion should be avoided and small angles of flexion (less than 30°) can be allowed in the period immediately following ACL reconstruction.

This study has several limitations. The entire range of motion was not studied. Hyperextension and flexion angles beyond 100° of flexion were not analysed. Only a lunge activity was used, and other functional activities, such as walking and ascending stairs, should be considered. Comparing the current results with the literature was not easy because there is no standard method for modelling and performing simulations. Therefore, the attachment points of the graft were identified by the bony landmarks individually. There was a concern that the radiation would be harmful to the subject. According to the record, the cumulative radiation dose was within the safety level and could be markedly reduced in future study [33]. Furthermore, the present study was conducted using data from a single subject. The same procedure should be repeated in other subjects to determine whether this is a common result.

## Conclusion

This study used dual fluoroscopic imaging and CT-based finite element modelling to estimate forces and stresses of grafts for anatomical single-bundle ACL reconstruction. The results of this study confirm that posterior to the centre of the femoral footprint is the strategic location for graft placement, and graft placement in this location results in anatomical graft behaviour with a low level of stress. This information may help surgeons optimize anatomical single-bundle ACL reconstruction or revision. Additional computational analyses and clinical studies are necessary to verify the practical significance of this conclusion.

## References

[1] Reynaud O, Batailler C, Lording T, Lustig S, Servien E, Neyret P (2017). Three dimensionalCT analysis of femoral tunnel position after ACL reconstruction. A prospective study of one hundred and thirty five cases. Int Orthop.

[2] Ferretti M, Ekdahl M, Shen W, Fu FH (2007). Osseous landmarks of the femoral attachment of the anterior cruciate ligament: an anatomic study. Arthroscopy.

[3] Shino K, Suzuki T, Iwahashi T, Mae T, Nakamura N, Nakata K, Nakagawa S (2010). The resident’s ridge as an arthroscopic landmark for anatomical femoral tunnel. Knee Surg Sports Traumatol Arthrosc.

[4] DeFrate LE (2017). Effects of ACL graft placement on in vivo knee function and cartilage thickness distributions. J Orthop Res.

[5] Markolf KL, Gorek JF, Kabo JM, Shapiro MS (1990). Direct measurement of resultant forces in the anterior cruciate ligament. An in vitro study performed with a new experimental technique. J Bone Joint Surg Am.

[6] Markolf KL, O'Neill G, Jackson SR, McAllister DR (2004). Effects of applied quadriceps and hamstrings muscle loads on forces in the anterior and posterior cruciate ligaments. Am J Sports Med.

[7] Kernkamp WA, Varady NH, Li JS, Tsai TY, Asnis PD, van Arkel ERA, Nelissen RGHH, Gill TJ, Van de Velde SK, Li G (2018). An in vivo prediction of anisometry and strain in anterior cruciate ligament reconstruction - a combined magnetic resonance and dual fluoroscopic imaging analysis. Arthroscopy.

[8] Chen B, Lambrou T, Offiah AC, Gondim Teixeira PA, Fry M, Todd-Pokropek A (2013). An in vivo subject-specific 3D functional knee joint model using combined MR imaging. Int J Comput Assist Radiol Surg.

[9] Charbonnier C, Duthon VB, Chagué S, Kolo FC, Ménétrey J (2020). In vivo static and dynamic lengthening measurements of the posterior cruciate ligament at high knee flexion angles. Int J Comput Assist Radiol Surg.

[10] Kernkamp WA, Wang C, Li C, Hu H, van Arkel ERA, Nelissen RGHH, LaPrade RF, van de Velde SK, Tsai TY (2019). The medial patellofemoral ligament is a dynamic and anisometric structure: an in vivo study on length changes and isometry. Am J Sports Med.

[11] Li G, Wuerz TH, DeFrate LE (2004). Feasibility of using orthogonal fluoroscopic images to measure in vivo joint kinematics. J Biomech Eng.

[12] Li G, Van de Velde SK, Bingham JT (2008). Validation of a non-invasive fluoroscopic imaging technique for the measurement of dynamic knee joint motion. J Biomech.

[13] Li JS, Tsai TY, Wang S, Li P, Kwon YM, Freiberg A, Rubash HE, Li G (2014). Prediction of in vivo knee joint kinematics using a combined dual fluoroscopy imaging and statistical shape modeling technique. J Biomech Eng.

[14] Chao EY, Laughman RK, Schneider E, Stauffer RN (1983). Normative data of knee joint motion and ground reaction forces in adult level walking. J Biomech.

[15] Bae JY, Kim GH, Seon JK, Jeon I (2016). Finite element study on the anatomic transtibial technique for single-bundle anterior cruciate ligament reconstruction. Med Biol Eng Comput.

[16] Pena E, Calvo B, Martinez MA, Doblare M (2006). A three-dimensional finite element analysis of the combined behavior of ligaments and menisci in the healthy human knee joint. J Biomech.

[17] Orsi AD, Chakravarthy S, Canavan PK, Peña E, Goebel R, Vaziri A, Nayeb-Hashemi H (2015). The effects of knee joint kinematics on anterior cruciate ligament injury and articular cartilage damage. Comput Methods Biomech Biomed Engin.

[18] Arneja S, McConkey MO, Mulpuri K, Chin P, Gilbart MK, Regan WD, Leith JM (2009). Graft tensioning in anterior cruciate ligament reconstruction: a systematic review of randomized controlled trials. Arthroscopy.

[19] Mae T, Shino K, Nakata K, Toritsuka Y, Otsubo H, Fujie H (2008). Optimization of graft fixation at the time of anterior cruciate ligament reconstruction. Part II: effect of knee flexion angle. Am J Sports Med.

[20] Orsi AD, Canavan PK, Vaziri A, Goebel R, Kapasi OA, Nayeb-Hashemi H (2017). The effects of graft size and insertion site location during anterior cruciate ligament reconstruction on intercondylar notch impingement. Knee.

[21] Wan C, Hao Z (2018). Does the graft-tunnel friction influence knee joint kinematics and biomechanics after anterior cruciate ligament reconstruction? A finite element study. Comput Methods Biomech Biomed Engin.

[22] Westermann RW, Wolf BR, Elkins JM (2013). Effect of ACL reconstruction graft size on simulated Lachman testing: a finite element analysis. Iowa Orthop J.

[23] Gasser TC, Ogden RW, Holzapfel GA (2006). Hyperelastic modelling of arterial layers with distributed collagen fibre orientations. J R Soc Interface.

[24] Suggs J, Wang C, Li G (2003). The effect of graft stiffness on knee joint biomechanics after ACL reconstruction–a 3D computational simulation. Clin Biomech.

[25] Ugur L (2017). Comparison of reaction forces on the anterior cruciate and anterolateral ligaments during internal rotation and anterior drawer forces at different flexion angles of the knee joint. Int J Med Robot.

[26] Laboureau JP, Marnat-Perrichet F, Yahia L (1997). Isometric reconstruction of the anterior cruciate ligament: femoral and tibial tunnel placement. Ligaments and ligamentoplasties.

[27] Rodeo SA, Kawamura S, Kim HJ, Dynybil C, Ying L (2006). Tendon healing in a bone tunnel differs at the tunnel entrance versus the tunnel exit: an effect of graft-tunnel motion?. Am J Sports Med.

[28] Beynnon BD, Uh BS, Johnson RJ, Fleming BC, Renström PA, Nichols CE (2001). The elongation behavior of the anterior cruciate ligament graft in vivo. A long-term follow-up study. Am J Sports Med.

[29] Arnold MP, Verdonschot N, van Kampen A (2005). The normal anterior cruciate ligament as a model for tensioning strategies in anterior cruciate ligament grafts. Am J Sports Med.

[30] Murray PJ, Alexander JW, Gold JE, Icenogle KD, Noble PC, Lowe WR (2010). Anatomic double-bundle anterior cruciate ligament reconstruction: kinematics and knee flexion angle-graft tension relation. Arthroscopy.

[31] Zavras TD, Race A, Amis AA (2005). The effect of femoral attachment location on anterior cruciate ligament reconstruction: graft tension patterns and restoration of normal anterior-posterior laxity patterns. Knee Surg Sports Traumatol Arthrosc.

[32] Kang K, Bae TS (2017). Effect of femoral tunnel positions on graft stress in outside-in ACL reconstruction surgery during continuous knee motion: a simulation study. Int J Med Robot.

[33] ICRP (2007). The 2007 recommendations of the international commission on radiological protection. ICRP publication 103. Ann ICRP.

